# Impact of Salt and Nutrient Content on Biofilm Formation by *Vibrio fischeri*

**DOI:** 10.1371/journal.pone.0169521

**Published:** 2017-01-25

**Authors:** Anne E. Marsden, Kevin Grudzinski, Jakob M. Ondrey, Cindy R. DeLoney-Marino, Karen L. Visick

**Affiliations:** 1 Department of Microbiology and Immunology, Loyola University Chicago, Maywood, Illinois, United States of America; 2 Department of Biology, University of Southern Indiana, Evansville, Indiana, United States of America; National Institute of Technology Rourkela, INDIA

## Abstract

*Vibrio fischeri*, a marine bacterium and symbiont of the Hawaiian bobtail squid *Euprymna scolopes*, depends on biofilm formation for successful colonization of the squid’s symbiotic light organ. Here, we investigated if culture conditions, such as nutrient and salt availability, affect biofilm formation by *V*. *fischeri* by testing the formation of wrinkled colonies on solid media. We found that *V*. *fischeri* forms colonies with more substantial wrinkling when grown on the nutrient-dense LBS medium containing NaCl relative to those formed on the more nutrient-poor, seawater-salt containing SWT medium. The presence of both tryptone and yeast extract was necessary for the production of “normal” wrinkled colonies; when grown on tryptone alone, the colonies displayed a divoting phenotype and were attached to the agar surface. We also found that the type and concentration of specific seawater salts influenced the timing of biofilm formation. Of the conditions assayed, wrinkled colony formation occurred earliest in LBS(-Tris) media containing 425 mM NaCl, 35 mM MgSO_4_, and 5 mM CaCl_2_. Pellicle formation, another measure of biofilm development, was also enhanced in these growth conditions. Therefore, both nutrient and salt availability contribute to *V*. *fischeri* biofilm formation. While growth was unaffected, these optimized conditions resulted in increased *syp* locus expression as measured by a P_*sypA*_*-lacZ* transcriptional reporter. We anticipate these studies will help us understand how the natural environment of *V*. *fischeri* affects its ability to form biofilms and, ultimately, colonize *E*. *scolopes*.

## Introduction

Growth within a biofilm offers many advantages to a bacterial cell and can be critical for survival in the face of unpredictable or harsh environmental conditions. For example, bacteria within biofilms are more resistant to antibiotics and host defenses due, in part, to the physical barrier provided by an extracellular matrix comprised of substances such as polysaccharides, proteins, and extracellular DNA [1]. For some organisms, biofilm formation is required for survival within and/or colonization of a host; thus, biofilms can be critical determinants of pathogenic and symbiotic bacteria-host interactions [2, 3].

*Vibrio fischeri*, a marine bacterium and symbiont of the Hawaiian bobtail squid *Euprymna scolopes*, depends on biofilm formation for successful colonization of the squid’s symbiotic light organ [4]. Although newly hatched *E*. *scolopes* are aposymbiotic, *V*. *fischeri* can be isolated from the light organ crypts of juvenile squid within hours of hatching [5]. This rapid association depends on the formation of a transient *V*. *fischeri* biofilm on the light organ surface; *V*. *fischeri* mutants unable to form biofilms do not enter or colonize the light organ crypts [4, 6]. *V*. *fischeri* biofilms can be visualized on the ciliated fields on the surface of the light organ by confocal microscopy [7]. Two distinct host-independent phenotypes can serve as quick and easy surrogates for assessing biofilm formation: wrinkled colony formation on solid media and pellicle formation in liquid media [4, 8, 9]. Using these phenotypes, a number of genes involved in biofilm formation and colonization of the squid have been identified [6, 10–17].

While wild-type *V*. *fischeri* readily forms biofilms on the *E*. *scolopes* light organ, biofilm formation in laboratory culture requires the genetic manipulation of one or more regulators of polysaccharide synthesis. Overexpression of one such regulator, the sensor kinase RscS, is sufficient to induce biofilm formation on plates or in liquid cultures [18]. RscS is a hybrid sensor kinase, and its overexpression results in a complex phosphorelay with a second sensor kinase and two response regulators [18–20]. One of these response regulators is SypG, a transcriptional regulator that directly activates the 18-gene *syp (sy*mbiosis *p*olysaccharide) locus. This locus encodes putative enzymes and other proteins predicted to produce and export a polysaccharide component (Syp PS) of the biofilm matrix required for host colonization, and disruption of this locus results in loss of both wrinkled colony and pellicle phenotypes [4, 6].

Although a number of genes involved in biofilm formation have been identified, little is known about the physiological conditions required for this phenomenon in laboratory culture or with respect to symbiosis; however, several seawater components are known to affect *V*. *fischeri* functions that are relevant to biofilm formation. For example, magnesium at environmentally relevant concentrations promotes *V*. *fischeri* flagellation and motility; calcium also promotes motility at concentrations <40 mM [21]. Here we report the observation that *V*. *fischeri* biofilms, as measured by wrinkled colony formation, are dramatically different in appearance depending on the composition of the growth medium, and we describe a systematic investigation of how growth in the presence of specific salts and nutrients affects biofilm formation by *V*. *fischeri*.

## Materials and Methods

### Bacterial strains and media

*V*. *fischeri* strains used for this study are listed in Table 1. Biofilm-competent strain KV4366 was derived from MJM1198 (IG [*glpR-rscS*]::Tn*5*) following serial passaging on agar media to lose the Tn*5* delivery vector pMJM30. This strain contains a Tn*5* insertion in the intergenic region between *glpR* and *rscS*, and was identified following Tn*5* mutagenesis to identify mutants capable of forming biofilms under laboratory conditions. [17]. *V*. *fischeri* strains were maintained on Luria-Bertani salt (LBS) agar plates without glycerol (1% tryptone, 0.5% yeast extract, 342 mM NaCl, and 50 mM Tris pH 7.5 with 1.5% agar for solid media), and tetracycline (5 μg/ml) was added as necessary [22]. *Escherichia coli* strains DH5α and π3813 carrying plasmids in Table 1 were used for conjugation and maintained on Luria-Bertani (LB) agar plates with kanamycin (50 μg/ml), ampicillin (100 μg/ml), and/or thymidine (300 μM) added as necessary [18, 23–25]. Additional media used in this study include SWT (0.5% tryptone, 0.3% yeast extract, 210 mM NaCl, 35 mM MgSO_4_, 7 mM CaCl_2_, and 7 mM KCl), LBS(-Tris) (1% tryptone, 0.5% yeast extract, and 342 mM NaCl), HTY-SW (1% tryptone, 0.5% yeast extract, 210 mM NaCl, 35 mM MgSO_4_, 7 mM CaCl_2_, and 7 mM KCl), LTY-NaCl (0.5% tryptone, 0.3% yeast extract, and 342 NaCl), and LBS-Opt (1% tryptone, 0.5% yeast extract, 425 mM NaCl, 35 mM MgSO_4_, and 5 mM CaCl_2_) (Table 2) [4]. Media containing titrations (0.25–1%) of tryptone or yeast extract were prepared with 342 mM NaCl. NaCl titrations (210–500 mM) were prepared with 1% tryptone and 0.5% yeast extract. Salt titrations for MgSO_4_ (0–100 mM), CaCl_2_ (0–10 mM), and KCl (0–50 mM) were prepared with 1% tryptone, 0.5% yeast extract, and 342 mM NaCl. Solid media were made with 1.5% agar. For consistency, solid media were made by pipetting 25 ml of liquid agar media into petri plates.

**Table 1:** *V*. *fischeri* strains and plasmids used in this study.

*S*train	Relevant characteristics	Reference
ES114	Wild-type *Vibrio fischeri*	[26]
KV4366	IG (*glpR-rscS*)::Tn5^a^	This study
KV3299	Δ*sypE*	[19]
KV4925	Δ*sypA* attTn7::P_*sypA*_*-lacZ*	This study
Plasmid	Relevant characteristics	Reference
pKV69	Vector; Cm^R^, Tet^R^	[23]
pKG11	pKV69 carrying *rscS*; Cm^R^, Tet^R^,	[18]
pEAH73	pKV69 carrying *sypG*; Cm^R^, Tet^R^	[19]
pEVS104	Conjugal helper plasmid (*tra trb*); Kan^R^	[25]

^a^IG (*glpR-rscS*), Intergenic region between *glpR* and *rscS*; this strain was derived from MJM1198 [17].

**Table 2:** Composition of media used in this work.

	LBS(-Tris)^a^	SWT	HTY-SW	LTY-NaCl	T-NaCl	YE-NaCl	LBS-Opt
Tryptone (%)	1	0.5	1	0.5	Various	-	1
Yeast extract (%)	0.5	0.3	0.5	0.3	-	Various	0.5
NaCl (mM)	342	210	210	342	342	342	425
MgSO_4_ (mM)	-	35	35	-	-	-	35
KCl (mM)	-	7	7	-	-	-	-
CaCl_2_ (mM)	-	7	7	-	-	-	5

^a^LBS normally contains 50 mM Tris, but for the purposes of this work this component was omitted, and this medium, referred to as LBS(-Tris), was used except where indicated.

### Wrinkled colony assay

*V*. *fischeri* strains were grown overnight at 28°C in LBS broth with appropriate antibiotics. The following day, cultures were diluted 1:100 in LBS and grown at 28°C with shaking. After reaching mid-log phase, cultures were diluted to an OD_600_ of 0.2. Cultures were spotted in 10 μl aliquots onto the appropriate solid media (as indicated in the figure legends) and grown at room temperature (approximately 24°C). Spotted cultures were imaged at the indicated times using a consistent magnification with a Zeiss Stemi 2000-C dissecting microscope. At the final time point colonies were disrupted by dragging a toothpick across the center of the colony to assess cohesiveness; this is a previously described method for detecting Syp polysaccharide production [16, 17]. The images presented here are representative of at least two independent experiments. Due to the variability between experiments, a difference of 2 hours or less was not considered significant when determining differences in time to initiation of wrinkling. Between replicates, the time to initiation of wrinkling by *V*. *fischeri* under any given condition was consistent relative to the different conditions being tested in each experiment.

### Growth curve

Wild-type *V*. *fischeri* ES114 and the biofilm-competent strain KV4366 were grown overnight at 28°C on LBS agar. The next day, cultures started from single colonies were grown overnight in LBS at 28°C with shaking. On the day of the assay, cultures were diluted to an OD_600_ of 0.05 in LBS(-Tris) or LBS-Opt and grown at 24°C or 28°C with shaking. The optical density was measured every hour until cultures entered stationary phase or until cell clumping became visible. The growth curve reported here is representative of three independent experiments.

### Transcriptional reporter assay

From single colonies, *V*. *fischeri* strains were grown overnight at 28°C with shaking in LBS with appropriate antibiotics. The following day, cultures were diluted to an OD_600_ of 0.05 in the appropriate media (as indicated in the figure legends) and grown at 24°C with shaking. Samples were collected after 24 hours and assayed for β-galactosidase activity as previously described [27]. Miller units were calculated and reported as the average of at least three independent experiments. Statistical significance was determined by unpaired Student’s t-test using GraphPad Prism, version 6.0g, for Mac OS X (GraphPad Software, La Jolla, CA).

### Pellicle assay

Cultures of *V*. *fischeri* grown overnight at 28°C in LBS were used to inoculate 2 ml of the appropriate growth media (indicated in the figure legends) at an OD_600_ of 0.3. Cultures were grown in duplicate in the center wells of a 24-well plate at 24°C and imaged with a Zeiss Stemi 2000-C dissecting microscope after 20, 24, and 48 hours (h). At each time point, the pellicles were disrupted with a toothpick to better visualize pellicle formation. Representative images are shown from at least two independent experiments.

## Results

### *V*. *fischeri* biofilm formation varies depending on the growth medium

**Fig 1:**
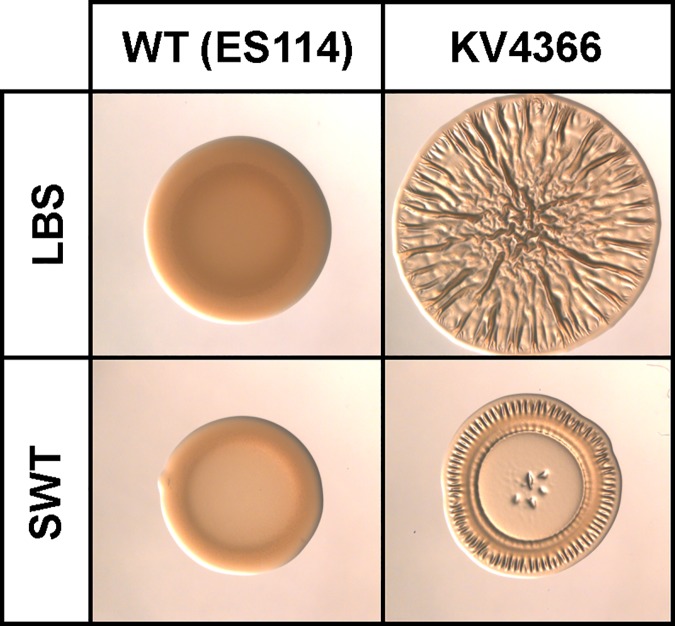
Wrinkled colony formation on two complex media, LBS and SWT. Cultures of ES114 (wild-type, non-biofilm induced) and KV4366 (RscS-overproducing, biofilm-induced) were grown in LBS and spotted onto LBS or SWT. Images were captured at the same magnification following growth for 48 h.

To better understand the environmental conditions that affect biofilm formation by *V*. *fischeri*, we investigated the ability of *V*. *fischeri* to form wrinkled colonies on a variety of complex solid media. We previously reported that wild-type *V*. *fischeri* strain ES114 forms smooth colonies on a complex solid medium (LBS), while RscS-overproducing, biofilm-competent derivatives, including KV4366, form wrinkled colonies (Fig 1) [17, 18, 28]. LBS contains tryptone, yeast extract, NaCl, and Tris buffer (pH 7.5) (Table 2) [29]. Another complex medium commonly used to grow *V*. *fischeri*, SWT, contains lower concentrations of tryptone and yeast extract and a mixture of salts (NaCl, MgSO_4_, CaCl_2_ and KCl) commonly found in seawater (Table 2). As expected, ES114 formed smooth colonies on SWT, while KV4366 formed wrinkled colonies; however, the colony morphology of KV4366 was noticeably different on SWT compared to LBS. Unlike the robust wrinkling displayed throughout the LBS-grown colony, wrinkling was primarily observed around the perimeter of the colony when grown on SWT (Fig 1). The inability of *V*. *fischeri* to form fully wrinkled colonies on SWT suggested that a component of SWT inhibits biofilm formation and/or a component of LBS is not present in sufficient concentrations in SWT to permit wrinkled colony formation. Therefore, the remainder of our work focused on defining the contributions of individual components of the two media, LBS and SWT, to promoting wrinkled colony formation by *V*. *fischeri*.

### Nutrient and salt concentrations contribute to biofilm formation

**Fig 2:**
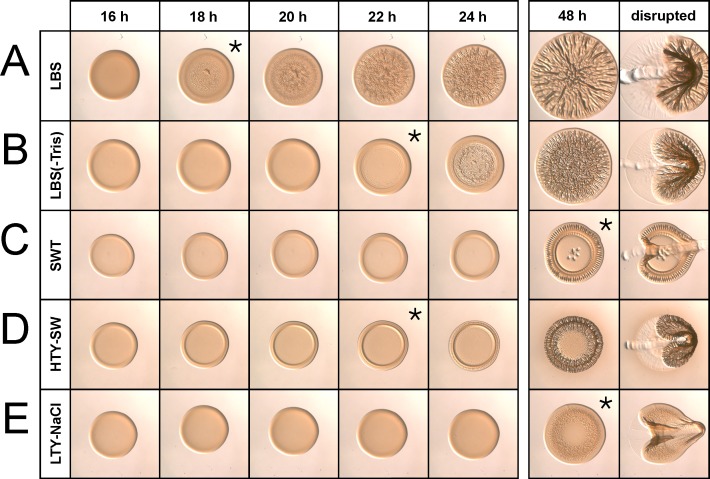
Impact of nutrients and seawater salts on wrinkled colony morphology. KV4366 was grown in LBS and spotted onto LBS (A), LBS(-Tris) (B), SWT (C), HTY-SW (D), or LTY-NaCl (E). Images were captured at the same magnification at the indicated time points. At the last time point, colonies were disturbed with a toothpick. The 48 h images for LBS and SWT are the same as those shown in Fig 1. Asterisks indicate the first time point at which wrinkling became visible.

To identify the components of LBS and SWT that account for the observed differences in colony phenotypes, we generated a panel of modified media (Table 2). First, we assessed the effect of Tris buffer on wrinkled colony formation and found that its absence from LBS medium [LBS(-Tris)] delayed the timing of wrinkled colony formation but did not substantially affect overall colony morphology, with wrinkling observed throughout both colonies (Fig 2A and 2B). Therefore, for simplicity, we omitted Tris buffer from subsequent experiments.

To assess the contribution of nutrient content on wrinkled colony formation, we combined the high nutrient content of LBS with the diversity of salts in SWT (210 mM NaCl, 35 mM MgSO_4_, 7 mM KCl, and 7 mM CaCl_2_) to make HTY-SW (high tryptone/yeast extract with seawater salts). Similarly, we combined the lower nutrient content of SWT with the salt composition of LBS (342 mM NaCl) to make LTY-NaCl (low tryptone/yeast extract with NaCl). We compared the development of KV4366 colony morphology on HTY-SW, LTY-NaCl, LBS(-Tris), and SWT (Fig 2). As observed previously, KV4366 formed colonies with extensive wrinkling or colonies with prominent ridges on the perimeter when grown on LBS(-Tris) or SWT, respectively (Fig 2B and 2C). Intriguingly, the patterns of biofilm formation by KV4366 on the modified media shared commonalities with both of the control media (Fig 2D and 2E). Specifically, wrinkled colony architecture developed primarily around the outside edges of the colony when grown on SWT and HTY-SW (Fig 2C and 2D), suggesting that a component of seawater promotes this phenotype. The colony architecture was also similar on LBS(-Tris) and LTY-NaCl, with centrally located wrinkling rather than peripheral ridges; however, the wrinkling on LTY-NaCl developed more slowly and remained reduced at 48 h, suggesting that the higher nutrient concentration in LBS(-Tris) contributes to earlier development of wrinkled colonies on this medium (Fig 2B and 2E).

To further assess biofilm development, we used a toothpick to disrupt the colonies. This assay identifies the production of Syp PS [16, 17], which is correlated with colony coherence (cells sticking to each other) and adherence (cells sticking to the agar). Both phenotypes are absent from colonies formed by ES114, which does not produce Syp PS in culture and fails to form wrinkled colonies (S6A Fig) [18]. KV4366 formed colonies with distinct properties depending on the medium. Colonies on LBS(-Tris) were coherent and could be pulled away from the agar intact, while the center of colonies on SWT adhered to the agar surface (Fig 2B and 2C). Colonies that formed on HTY-SW and LTY-NaCl most closely resembled those that formed on LBS(-Tris) and SWT, respectively (Fig 2B–2E). Together, these data indicate that the same *V*. *fischeri* strain can exhibit different biofilm phenotypes, depending on the medium. Furthermore, nutrient concentration appears to be a determinant of coherent versus adherent phenotypes; high nutrient content (LBS[-Tris] or HTY-SW) promotes the development of coherent colonies, while low nutrient content (SWT or LTY-NaCl) promotes adherent colony development (Fig 2B–2E).

### The role of tryptone and yeast extract in wrinkled colony formation

**Fig 3:**
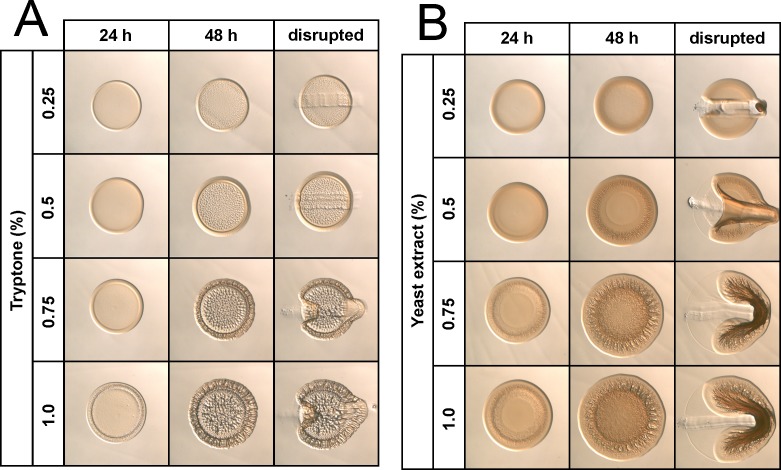
Impact of tryptone and yeast extract on wrinkled colony morphology. KV4366 was grown in LBS and spotted onto medium containing only 342 mM NaCl and tryptone (A) or yeast extract (B) at amounts ranging from 0.25–1.0%, as indicated. Images were captured at the same magnification after 24 or 48 h, and at the last time point colonies were disturbed with a toothpick.

To better assess the roles of the two nutrient sources, tryptone and yeast extract, in the wrinkled colony phenotype, we made media containing NaCl (342 mM) and increasing amounts (0.25 to 1.0%) of either tryptone or yeast extract as the sole nutrient source (referred to as T-NaCl and YE-NaCl; Table 2). When tryptone was provided as the sole nutrient source, development of colony architecture was delayed compared to growth on LBS(-Tris) (compare 24 h time points for Figs 2B and 3A). Additionally, colonies grown on T-NaCl appeared to form depressions or “divot” into the agar, rather than form the wrinkles that are observed on LBS(-Tris) (S1 Fig). Upon closer inspection, divoting was also found to occur with growth on SWT (Fig 2C, 48 h time point). To our knowledge, this is the first description of this phenotype for *V*. *fischeri*. The addition of 0.75–1.0% tryptone induced wrinkling around the colony perimeter and increased divoting within the colony center (Fig 3A). All colonies grown on T-NaCl were adherent to the agar surface; however, high amounts of tryptone relieved adherence at the edges of the colony. We conclude that tryptone promotes the ability of *V*. *fischeri* to adhere to the agar surface and to form a divoting colony architecture.

When yeast extract was provided as the sole nutrient source, colony architecture varied depending on the amount of yeast extract added (Fig 3B). At lower amounts of yeast extract (0.25%), colonies did not wrinkle or adhere to the agar surface. With increasing concentrations of yeast extract (0.5%), wrinkles began to develop, and colonies were adherent to the agar surface. Finally, at the highest concentrations of yeast extract (0.75–1.0%), colonies exhibited a wrinkled phenotype and were largely coherent. However, compared to growth on LBS(-Tris), wrinkled colony formation was not as well-defined with yeast extract alone, suggesting that the overall higher nutrient content in LBS(-Tris) or a specific component of tryptone is necessary for this robust phenotype. Overall, we conclude that increasing concentrations of yeast extract promote wrinkled colony formation and colony cohesiveness.

Together, these experiments indicate that even relatively low amounts of nutrients are sufficient to promote Syp PS production, as indicated by the agar adherence phenotype (Fig 3). They also suggest that a component of yeast extract, when present in sufficient amounts, results in the loss of adherence. Finally, these data indicate that tryptone must also make a positive contribution to the cohesive phenotype, as yeast extract, when present as the sole nutrient at concentrations similar to those present in LBS (0.5%), promoted adherence rather than coherence (Fig 3B).

### Wrinkled colony formation is sensitive to salt concentrations

**Fig 4:**
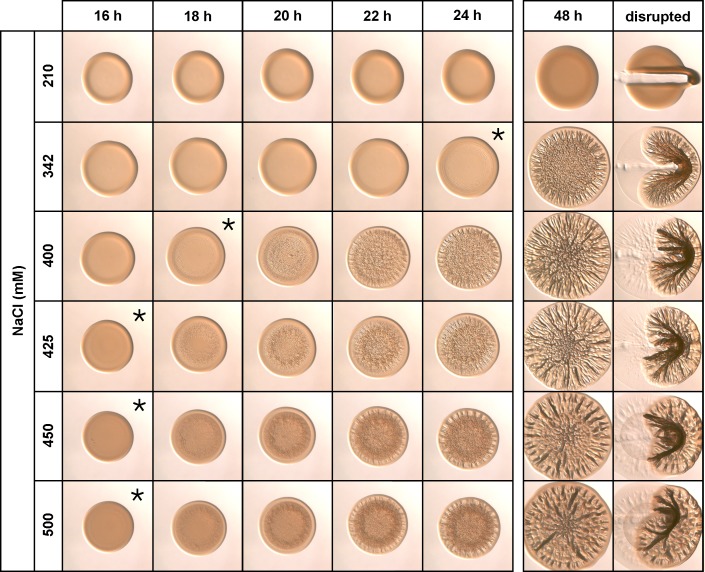
Sodium chloride promotes wrinkled colony morphology. KV4366 was grown in LBS and spotted onto medium containing 1.0% tryptone, 0.5% yeast extract, and 210–500 mM NaCl as indicated. Colonies were imaged at the same magnification at the indicated time points and disturbed with a toothpick at the last time point. Asterisks indicate the first time point at which wrinkling became visible.

We next assessed the role of salts in the ability of *V*. *fischeri* to form wrinkled colonies. The concentration of NaCl differs between LBS and SWT (342 mM and 210 mM, respectively). Therefore, we asked what concentration of NaCl was sufficient to promote wrinkled colony formation. When provided with the nutrient content used in LBS, 210 mM NaCl did not promote colony wrinkling, adherence, or cohesion within 48 h (Fig 4). When provided in higher concentrations, we found that NaCl up to 500 mM decreased the time to the start of wrinkled colony formation by 6 to 8 h compared to growth on LBS(-Tris) (Figs 4 and 2B). We conclude that NaCl promotes wrinkled colony formation, and the amount of NaCl in LBS (342 mM), but not SWT (210 mM), is sufficient for robust biofilm formation by *V*. *fischeri* within 48 h.

**Fig 5:**
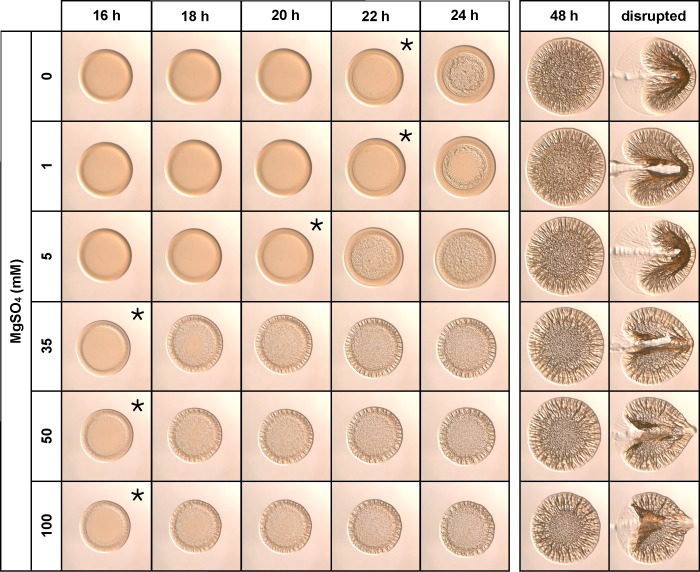
Magnesium sulfate promotes wrinkled colony morphology. KV4366 was grown in LBS and spotted onto medium containing 1.0% tryptone, 0.5% yeast extract, 342 mM NaCl, and 0–100 mM MgSO_4_. Colonies were imaged at the same magnification at the indicated times and disturbed with a toothpick at the last time point. Asterisks indicate the first time point at which wrinkling became visible.

To examine the role of the additional salts present in SWT, wrinkled colony formation was assessed in the presence of increasing concentrations of MgSO_4_, KCl, and CaCl_2_. Following NaCl, MgSO_4_ is present at the highest concentration (35 mM) in SWT. The addition of 35 mM MgSO_4_ to LBS(-Tris) resulted in a reproducibly earlier initiation of wrinkled colony development (two to three hours sooner than in its absence) (Fig 5). Furthermore, the presence of MgSO_4_ consistently led to distinct wrinkling at the perimeter of the colony, a phenotype not seen on LBS(-Tris) nor upon the addition of other salts. To examine this effect further, we assessed wrinkled colony formation following addition of 1–100 mM MgSO_4_. Concentrations <5 mM MgSO_4_ exerted little effect on the timing of wrinkled colony formation, but concentrations ≥5 mM promoted early development of wrinkled colonies. Perimeter wrinkling was evident when MgSO_4_ was provided at concentrations of 35, 50, or 100 mM. This phenotype is also present when MgSO_4_ is substituted with MgCl_2_, suggesting that Mg^2+^, rather than SO_4_^2-^ or Cl^-^, influences the formation of perimeter wrinkling (S2 Fig). We conclude that MgSO_4_, when present in higher concentrations, promotes the development of wrinkled colonies. Additionally, of the concentrations tested, 50 and 100 mM MgSO_4_ also resulted in increased agar adherence, another phenotype that is characteristic of growth on SWT (Figs 2C and 5).

**Fig 6:**
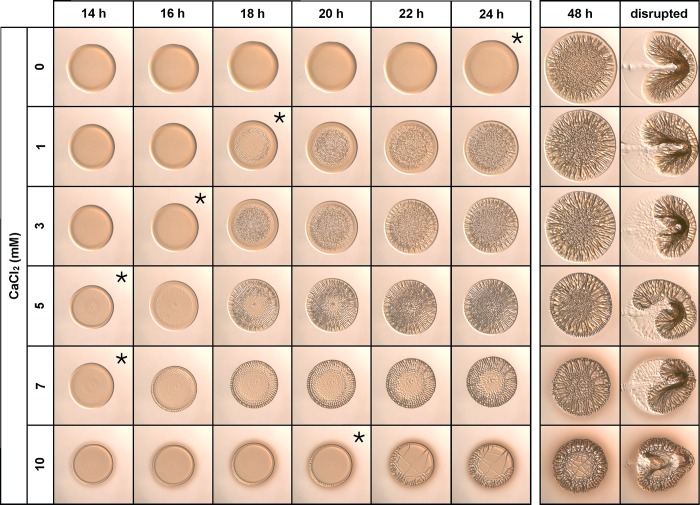
Calcium chloride promotes wrinkled colony morphology. KV4366 was grown in LBS and spotted onto medium containing 1.0% tryptone, 0.5% yeast extract, 342 mM NaCl, and 0–10 mM CaCl_2_. Colonies were imaged at the same magnification at the indicated times and disturbed with a toothpick at the last time point. Asterisks indicate the first time point at which wrinkling became visible.

Calcium is known to promote biofilm production in many bacteria, and the presence of CaCl_2_ in SWT prompted us to examine its role in *V*. *fischeri* biofilm formation [30–32]. When KV4366 was grown with CaCl_2_, we observed a substantial positive effect on wrinkled colony development (Fig 6). This effect was most apparent with 5 mM CaCl_2_, which promoted wrinkling after only 14 h of growth, compared to wrinkled colony development after 20 h of growth on LBS(-Tris) (Figs 6 and 2B). In contrast, the addition of 10 mM CaCl_2_ to LBS(-Tris) inhibited the development of wrinkles, and the colony formed a flat surface with a cracked appearance. We conclude that, under these conditions, 5 mM CaCl_2_ was optimal in promoting wrinkled colony formation.

KCl is present in SWT at a concentration of 7 mM; however, addition of 7 mM KCl to LBS(-Tris) exerted little impact on wrinkled colony development (S3 Fig). Higher concentrations of KCl (14–50 mM) accelerated wrinkled colony formation, but only when present at concentrations greater than that found in SWT. Thus, KCl can influence biofilm development when present at a sufficiently high concentration, but the colony phenotype observed on LBS(-Tris) with KCl concentrations normally present in SWT appears to be KCl-independent.

### LBS and SWT salt components promote *syp* locus transcription

**Fig 7:**
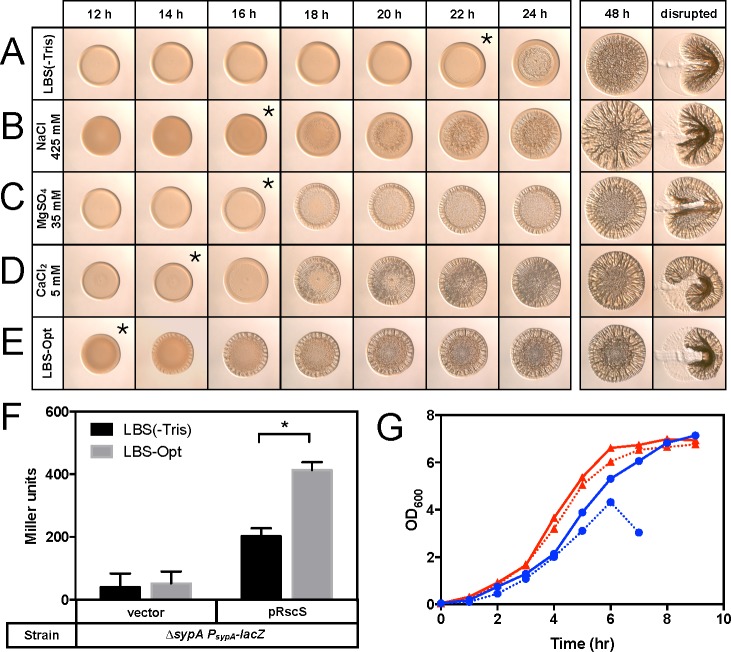
Optimal media promotes wrinkled colony morphology and *syp* locus transcription. KV4366 was grown in LBS and spotted onto LBS(-Tris) (A) or LBS(-Tris) with 425 mM NaCl (B), 35 mM MgSO_4_ (C), 5 mM CaCl_2_ (D), or LBS-Opt (E). Images were captured at the same magnification at the indicated times, and asterisks indicate the time at which wrinkling became visible. At the last time point colonies were disturbed with a toothpick. Images in parts A-D are selections from Figs 2, 4, 5 and 6. To measure *syp* locus transcription, Δ*sypA* mutant strains carrying a P_*sypA-lacZ*_ transcriptional reporter and a plasmid overproducing RscS (pKG11/Δ*sypA*) or vector control (pKV69/Δ*sypA*) were grown in LBS(-Tris) or LBS-Opt at 24°C for 24 h and assayed for β-galactosidase activity (F). Error bars represent the standard deviation of at least three biological replicates. *, *P*<0.05. To assess the effect of LBS-OPT on growth, KV4366 was grown with shaking at 24°C (blue circles) and 28°C (red triangles) in LBS(-Tris) (solid line) or LBS-Opt (dotted line), and the absorbance at 600 nm was measured each hour (G).

Based on our observations of enhanced wrinkled colony formation when grown with the individual salt components of LBS and SWT, we generated a medium composed of LBS(-Tris) with 425 mM NaCl, 35 mM MgSO_4_, and 5 mM CaCl_2_. We have designated this medium LBS-Opt to indicate that it contains the optimal concentration of each salt when tested individually (*i*.*e*., the salt concentrations that promoted the earliest formation of wrinkled colonies). We then compared biofilm formation on LBS-Opt compared to LBS(-Tris) alone and with each individual salt. Growth on LBS-Opt resulted in evidence of wrinkling after only 12 h (Fig 7E). Of the individual components of LBS-Opt, CaCl_2_ had the most conspicuous effect on the timing and structure of wrinkled colony development; addition of CaCl_2_ alone resulted in a phenotype most similar to that formed on LBS-Opt, with only a slight delay in appearance of wrinkling compared to growth on LBS-Opt (Fig 7D).

Due to the lack of a known signal for inducing *V*. *fischeri* biofilm formation in laboratory conditions, wrinkled colony studies are performed with biofilm competent strains that overexpress positive regulators of the *syp* locus. KV4366 contains a chromosomal insertion that results in the overproduction of RscS, but many studies have been performed using plasmids that overproduce RscS or the direct transcriptional activator SypG to induce biofilm formation [4, 18, 20, 33]. Thus, we asked if the LBS-Opt medium could also promote wrinkling by other biofilm competent strains, *i*.*e*., those carrying plasmids to induce biofilm formation. Although the final appearance of wrinkled colonies was altered, growth on LBS-Opt and the individual components of LBS-Opt resulted in early development of wrinkling compared to LBS(-Tris), suggesting that the stimulatory effect of LBS-Opt is not specific to strain KV4366 (S4 Fig). In contrast, despite the stimulatory effect of LBS-Opt for biofilm-competent strains, it was not sufficient to promote wrinkled colony formation by wild-type ES114, suggesting LBS-Opt does not provide the necessary signal or combination of factors required to activate biofilm production by wild-type *V*. *fischeri* (S6A Fig). Interestingly, when grown on 5 mM CaCl_2_, wild-type ES114 (non-biofilm induced) appeared to be very modestly coherent, as evidenced by the jagged edge where the colony was disrupted after 48 h of growth (S6A Fig).

*V*. *fischeri* biofilm formation requires transcription of the *syp* locus and subsequent production of Syp PS [4]. Increased activation of this locus during growth on LBS-Opt could account for the enhanced wrinkled colony formation observed on this media. To examine the effect of LBS-Opt on *syp* locus activity, we introduced an RscS overexpression vector into a Δ*sypA* mutant carrying a P_*sypA*_*-lacZ* transcriptional reporter and compared reporter activity in LBS(-Tris) and LBS-Opt. This background was chosen to eliminate the complications of Syp PS-mediated aggregation, as the Δ*sypA* mutant does not produce Syp PS [34]. Whereas reporter activity from the vector control did not significantly differ when grown in LBS(-Tris) or LBS-Opt, we observed a significant increase in reporter activity from the RscS-overexpressing strain grown in LBS-Opt compared to LBS(-Tris) (Fig 7F). These findings suggest that one or more of the salt components of LBS-Opt affect *syp* transcription.

A simple explanation for the earlier formation of wrinkled colonies and increase in *syp* locus transcription by biofilm competent strains in the presence of LBS-Opt is that this medium promotes better growth of *V*. *fischeri* than LBS(-Tris). To test this hypothesis, we measured the growth of biofilm competent KV4366 over time when grown in LBS(-Tris) and LBS-Opt at 24°C (Fig 7G). KV4366 exhibited minimal differences in optical density when grown in LBS(-Tris) or LBS-Opt. After six hours of growth, the KV4366 culture grown in LBS-Opt began to clump, consistent with the production of Syp PS due to RscS overproduction. As a result, an accurate measure of the optical density could not be obtained after the six-hour time point. RscS-overproducing strains promote Syp PS production at 24°C but not 28°C, the standard temperature used for laboratory culture of *V*. *fischeri* [33]. Therefore, to assess the effect of LBS-Opt on *V*. *fischeri* growth in the absence of Syp PS, we repeated this growth assay at 28°C. Again, very little difference in growth was observed for KV4366 grown in LBS(-Tris) or LBS-Opt. This observation suggests that LBS-Opt does not confer a growth advantage in liquid culture, and early wrinkled colony formation observed on LBS-Opt or its components is not solely due to faster bacterial growth.

### Calcium chloride and sodium chloride promote *V*. *fischeri* pellicle formation

**Fig 8:**
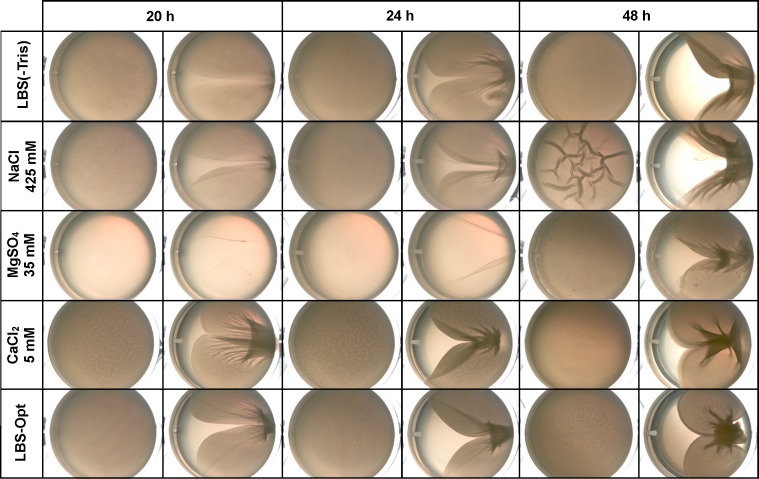
Impact of salts on *V*. *fischeri* pellicle formation. KV4366 was grown in LBS and diluted to an OD_600_ of 0.3 in LBS(-Tris) and LBS(-Tris) with 425 mM NaCl, 35 mM MgSO4, or 5 mM CaCl2, and LBS-Opt. Triplicate cultures were grown statically in the center wells of a 24-well plate at 24°C and imaged after 20, 24, and 48 h. One sample of the triple cultures was disrupted with a toothpick at each of the three time points; for each time point, images of the undisrupted and disrupted pellicles are presented from left to right.

In addition to wrinkled colonies, biofilm-induced *V*. *fischeri* cells can form pellicles at the air-liquid interface of static cultures. This measure of biofilm production also requires Syp PS, so we hypothesized that the components of LBS-Opt would also promote pellicle formation. When grown in liquid LBS(-Tris), static cultures of KV4366 formed visible pellicles within 20 h (Fig 8). Growth in LBS-Opt also promoted pellicle formation within 20 h; moreover, using a qualitative measure of biofilm thickness (disruption with a toothpick), the pellicle formed in LBS-Opt appeared to be thicker than pellicles formed in LBS(-Tris) after 20 h. Additionally, growth in LBS-Opt promoted the formation of wrinkles on the pellicle surface (S5 Fig). To determine how individual components of LBS-Opt affect pellicle formation, we added additional NaCl, MgSO_4_, or CaCl_2_ to LBS(-Tris) and imaged pellicles after 20, 24, and 48 h (Fig 8 and S5 Fig). The addition of 5 mM CaCl_2_ promoted pellicle formation and wrinkling to a similar degree as LBS-Opt within 20 h. Additional NaCl (425 mM) resulted in large wrinkles not present on pellicles grown on LBS(-Tris) after 48 h (S5 Fig). It is unclear whether the large wrinkles represent more or less Syp PS production than observed with LBS-Opt and LBS(-Tris) with 5 mM CaCl_2_, which both promote the production of small but dense wrinkles (S5 Fig). Another possibility is that CaCl_2_ or MgSO_4_ inhibits the production of large wrinkles during growth in LBS-Opt. Compared to CaCl_2_ or NaCl, pellicle formation with 35 mM MgSO_4_ was delayed and did not exhibit surface wrinkling. Based on these findings we conclude that, of the salts present in LBS and SWT, CaCl_2_ and NaCl promote biofilm production resulting in both wrinkled colonies and pellicle formation. As observed for the wrinkled colony phenotype, LBS-Opt media was not sufficient to promote pellicle formation by wild-type ES114, again highlighting the lack of stimulatory signal or factors required for biofilm production in the absence of RscS overexpression (S6B Fig). However, consistent with the low level of colony cohesion observed with CaCl_2_ alone (S6A Fig), a faint pellicle formed by ES114 was apparent after 48 h of growth in 5 mM CaCl_2_ liquid media (S6B Fig).

## Discussion

Biofilm formation by *V*. *fischeri* requires activation of the 18-gene *syp* locus and subsequent production of the Syp polysaccharide; therefore, the transition from a planktonic to biofilm lifestyle must be tightly controlled to be selectively beneficial. Signals controlling biofilm formation must exist to appropriately promote or inhibit this lifestyle depending on the environmental conditions. In this study, we evaluated the impact on biofilm formation of specific components of two media commonly used to culture *V*. *fischeri*, LBS and SWT. Our investigation of these media components revealed that the nutrient content and concentration as well as the presence of specific salts impact the ability of *V*. *fischeri* to form a biofilm.

Both LBS and SWT contain tryptone and yeast extract, albeit at different amounts (Table 2). Each of these complex nutrient mixtures elicited different dose-dependent effects on colony morphology. When provided as the sole nutrient source, yeast extract induced the appearance of colonies that were similar in appearance to those formed on LBS. In contrast, tryptone induced divoting, a previously undescribed phenotype for *V*. *fischeri* in which sections of the colony pressed down into the agar medium rather than upwards in the form of a wrinkle. Interestingly, these nutrient mixtures had variable effects on colony coherence and adherence. Divoted colonies that formed on tryptone were invariably attached to the agar surface, while yeast extract promoted both adherence and coherence depending on the concentration. A simple explanation for this observation is that increasing levels of Syp PS production result in a transition from adherent to coherent colonies. In support of this hypothesis is the progression from non-coherent/non-adherent to adherent and then to coherent with increasing concentrations of yeast extract (Fig 3B). Tryptone and yeast extract are both composed of complex mixtures of amino acids, vitamins, and carbohydrates in varied amounts. The concentration of many components differs significantly between the two nutrient sources. For example, the content of carbohydrates and certain vitamins is higher in yeast extract than tryptone. Therefore, another possibility to explain the formation of adherent and coherent colonies is that a component present in higher concentrations in yeast extract promotes the formation of cohesive colonies (and prevents divoting). The consequences of agar adherence and divoting on the physiology of the cells and the other factors involved in this process (other than Syp PS) are important areas of future investigation.

Our examination of the contribution of LBS and SWT salt components to wrinkled colony formation provided more insight into the specific requirements for this phenotype. NaCl, MgSO_4_, CaCl_2_, and KCl caused dose-dependent enhancement of wrinkled colony formation by reducing the time to onset of colony wrinkling. Considering the natural environment of *V*. *fischeri*, Na^+^, Mg^2+^, SO_4_^2-^, Ca^2+^, K^+^ and Cl^-^ are all major constituents of surface seawater. Except for K^+^, the reported concentrations of these substances are similar to those used to generate LBS-Opt (*i*.*e*., 481 mM Na^+^, 54 mM Mg^2+^, 29 mM SO_4_^2-^, 11 mM Ca^2+^, 559 mM Cl^-^ in surface seawater) [35]. While K^+^ is present in surface seawater at a concentration of approximately 10 mM, compared to the other salt components, the addition of 7 mM KCl or even a much higher concentration of KCl (50 mM) did not substantially decrease time to wrinkled colony formation (S3 Fig). For this reason, KCl was not included in the LBS-Opt media.

The variable concentrations of individual salts needed for the observed effects suggest that increased osmolarity is not the sole factor mediating wrinkled colony formation (*e*.*g*., 5 mM CaCl_2_ had a strong positive effect, but 7 mM KCl had very little effect). To begin to understand how *V*. *fischeri* biofilm formation is affected by growth in LBS-Opt media, we evaluated *syp* locus transcription and found that LBS-Opt significantly increased *syp* locus transcription relative to LBS(-Tris) (Fig 7F). This stimulatory effect can be attributed to the salt components of LBS-Opt, as the nutrient content (*i*.*e*., tryptone and yeast extract) in these media is equivalent; however, it remains unknown whether this effect is the result of one or more of these salts. Similarly, in *V*. *vulnificus*, the polysaccharide biosynthetic locus homologous to *syp* is upregulated in artificial seawater (when compared to human serum), suggesting there may be a common mechanism involving seawater salts that governs *syp* locus activation in some *Vibrio* spp. [36].

Several notable salt-specific phenotypes were observed. In addition to the effect on timing, we found that MgSO_4_ resulted in enhanced wrinkling at the colony perimeter, a phenotype characteristic of growth on SWT, suggesting that MgSO_4_ may be the SWT component responsible for this unique phenotype. The effect of CaCl_2_ on biofilm formation was perhaps the most notable. Compared to NaCl or MgSO_4_, addition of CaCl_2_ resulted in earlier wrinkled colony formation and promoted the development of pellicles that were indistinguishable from those formed on LBS-Opt (Fig 8). Additionally, a jagged edge was observed following disruption of wild-type ES114 (with and without empty vector) when grown on 5 mM CaCl_2_ (S4C, S4D and S6A Figs). Of the many conditions tested, only CaCl_2_ resulted in altered morphology for ES114 colonies. The basis for this morphology change is currently unknown, but may be indicative of Syp PS production below the threshold necessary for wrinkled colony formation or, alternatively, the production of another adherence factor such as cellulose [37]. Furthermore, the reason this phenotype is absent when ES114 is grown on LBS-Opt suggests a component of this medium may interfere with the CaCl_2_-mediated effect.

The effects of calcium on biofilm formation have been documented in a variety of bacteria, including *Vibrio* species. These include both positive and negative regulatory effects and roles as extracellular matrix components. For example, calcium inhibits the phosphodiesterase activity of the EAL domain protein VieA in *Vibrio cholerae*, preventing the hydrolysis of c-di-GMP, which is required for VPS (*Vibrio* polysaccharide)-dependent biofilm formation [38, 39]. VPS-independent biofilms have also been described for *V*. *cholerae* in which cell aggregation depends on the lipopolysaccharide O-antigen and the presence of Ca^2+^ [40]. Additionally, expression of the two-component regulatory system CarSR, which negatively regulates *V*. *cholerae* biofilm formation, is decreased in the presence of Ca^2+^[41]. *V*. *vulnificus* biofilm formation is also affected by calcium; the calcium-binding protein CabA is a matrix-associated protein. In this case, calcium-binding by CabA induces a conformational change thought to be required for incorporation and assembly of the biofilm matrix [32]. These examples of calcium-mediated effects demonstrate the broad range of mechanisms by which calcium can affect biofilm formation in *V cholerae and V*. *vulnificus*. Calcium clearly also affects biofilm formation by *V*. *fischeri*, but the mechanism through which it acts is unknown and will be the topic of further investigation.

One unexpected observation was the apparent differential regulation of wrinkled colonies and pellicles by the salt components of LBS-Opt. Specifically, while all three salts promoted wrinkled colony formation, the pellicles formed with MgSO_4_, CaCl_2_, and NaCl were dissimilar (Fig 8). One potential explanation is that the development of wrinkled colonies and pellicles requires overlapping but distinct sets of determinants. For example, Syp PS is a known component of both *V*. *fischeri* wrinkled colony and pellicle biofilms; however some evidence suggests that a second polysaccharide, cellulose, also contributes to biofilm formation under some conditions [13]. These observations support the possibility that the wrinkled colony and pellicle phenotypes do not require the same set of determinants.

Our understanding of the intricate nature of *V*. *fischeri* biofilm regulation is constantly evolving, but would suggest that ascribing the role of LBS-Opt solely to *syp* locus transcription is too simplistic; this complex media likely affects multiple factors that govern biofilm production. Also, biofilms that manifest as wrinkled colonies or pellicles may be differentially affected by these factors. Although the mechanism for LBS-Opt mediated *syp* locus activation and biofilm formation is unknown, this work highlights new avenues of investigation into the regulation of *V*. *fischeri* biofilms. Future studies will be performed to understand the differences between wrinkled colony and pellicle biofilms and to determine the role and specific mechanism for NaCl, MgSO_4_, and CaCl_2_ in *V*. *fischeri* biofilms formed under laboratory conditions and ultimately in the natural host, *E*. *scolopes*.

## Supporting Information

S1 Fig Impact of tryptone on colony divoting. KV4366 was grown in LBS and spotted onto medium containing 1% tryptone and 2% NaCl. Colonies were imaged at the same magnification at 24 and 48 h. After 48 h, the colony was disrupted with a toothpick. These images are the same as those in Fig 3 but are cropped and enlarged here to permit better visualization of the divoting phenotype.
(TIFF)

S2 Fig Impact of MgCl_2_ on colony morphology. KV4366 was grown in LBS and spotted onto medium containing 1.0% tryptone, 0.5% yeast extract, 342 mM NaCl, and 0–50 mM KCl. Colonies were imaged at the same magnification at the indicated times and disturbed with a toothpick at the last time point. Asterisks indicate the first time point at which wrinkling became visible.
(TIFF)

S3 Fig Impact of KCl on colony morphology. KV4366 was grown in LBS and spotted onto medium containing 1.0% tryptone, 0.5% yeast extract, 342 mM NaCl, and 0–50 mM KCl. Colonies were imaged at the same magnification at the indicated times and disturbed with a toothpick at the last time point. Asterisks indicate the first time point at which wrinkling became visible.
(TIFF)

S4 Fig Optimal media promotes wrinkled colony morphology by biofilm competent strains. Plasmid-containing strains were grown in LBS and spotted onto LBS(-Tris), LBS-Opt, or LBS(-Tris) with 425 mM NaCl, 35 mM MgSO_4_, or 5 mM CaCl_2_. Strains carried either biofilm-inducing plasmids overproducing RscS (pKG11/ES114) (A) or SypG (pEAH73/Δ*sypE*) (B) or contained vector control plasmid pKV69 (pKV69/ES114 and pKV69/Δ*sypE*) (C and D, respectively). Images were captured at the same magnification after 24 or 48 h of growth at 24°C. Colonies were disturbed with a toothpick at the 48 hour time point.
(TIFF)

S5 Fig Impact of salts on *V*. *fischeri* pellicle formation. KV4366 was grown in LBS and diluted to an OD_600_ of 0.3 in LBS(-Tris) and LBS(-Tris) with 425 mM NaCl, 35 mM MgSO4, or 5 mM CaCl2, and LBS-Opt. Cultures were grown statically in the center wells of a 24-well plate at 24°C and imaged after 48 h. These images are the same as those in Fig 8 but are enlarged here to permit better visualization of the pellicle morphology.
(TIFF)

S6 Fig Optimal media does not promote biofilm formation by wild-type *V*. *fischeri*. Wild-type *V*. *fischeri* strain ES114 was (A) grown in LBS and spotted onto LBS(-Tris) or LBS(-Tris) with 425 mM NaCl, 35 mM MgSO_4_, or 5 mM CaCl_2_, and LBS-Opt. Colonies were imaged at the same magnification after 24 or 48 h of growth at 24°C, and at the 48 hour time point colonies were disturbed with a toothpick. (B) ES114 was grown in LBS and diluted to an OD_600_ of 0.3 in LBS(-Tris) and LBS(-Tris) with 425 mM NaCl, 35 mM MgSO4, or 5 mM CaCl2, and LBS-Opt. Triplicate cultures were grown statically in the center wells of a 24-well plate at 24°C. After 20, 24, and 48 h, the surface of the liquid of each culture was disrupted with a toothpick and imaged; for each time point, images of the undisrupted and disrupted pellicles are presented from left to right. No pellicles were visualized.
(TIFF)
