# Supplementary figures and images for: Impact of Salt and Nutrient Content on Biofilm Formation by Vibrio fischeri
Source: PLoS One. 2017 Jan 25;12(1):e0169521. doi: 10.1371/journal.pone.0169521 (PMC5266276; doi:10.1371/journal.pone.0169521)

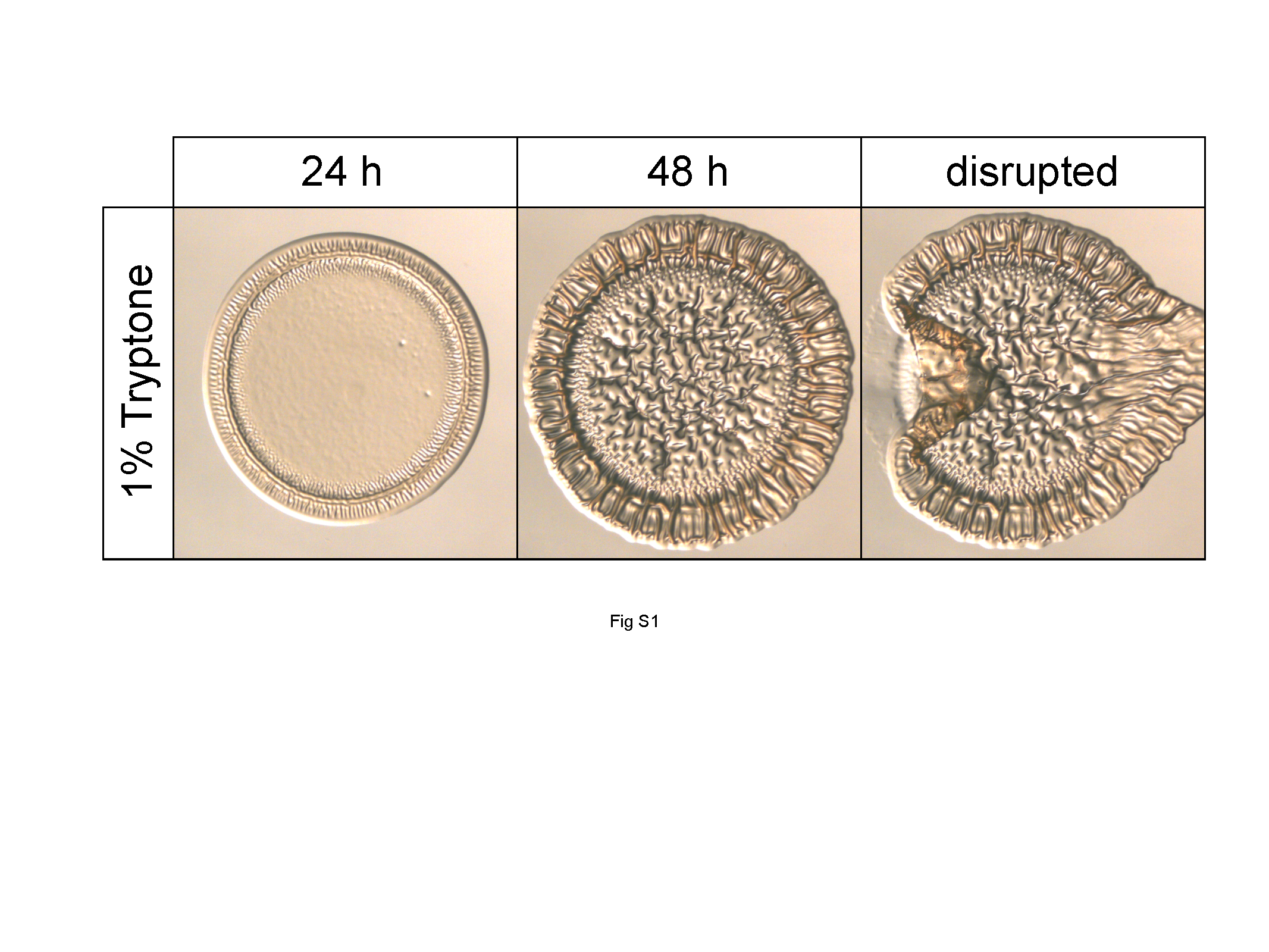

Supplement: S1 Fig — KV4366 was grown in LBS and spotted onto medium containing 1% tryptone and 2% NaCl. Colonies were imaged at the same magnification at 24 and 48 h. After 48 h, the colony was disrupted with a toothpick. These images are the same as those in Fig 3 but are cropped and enlarged here to permit better visualization of the divoting phenotype. (TIFF) [file pone.0169521.s001.tiff]

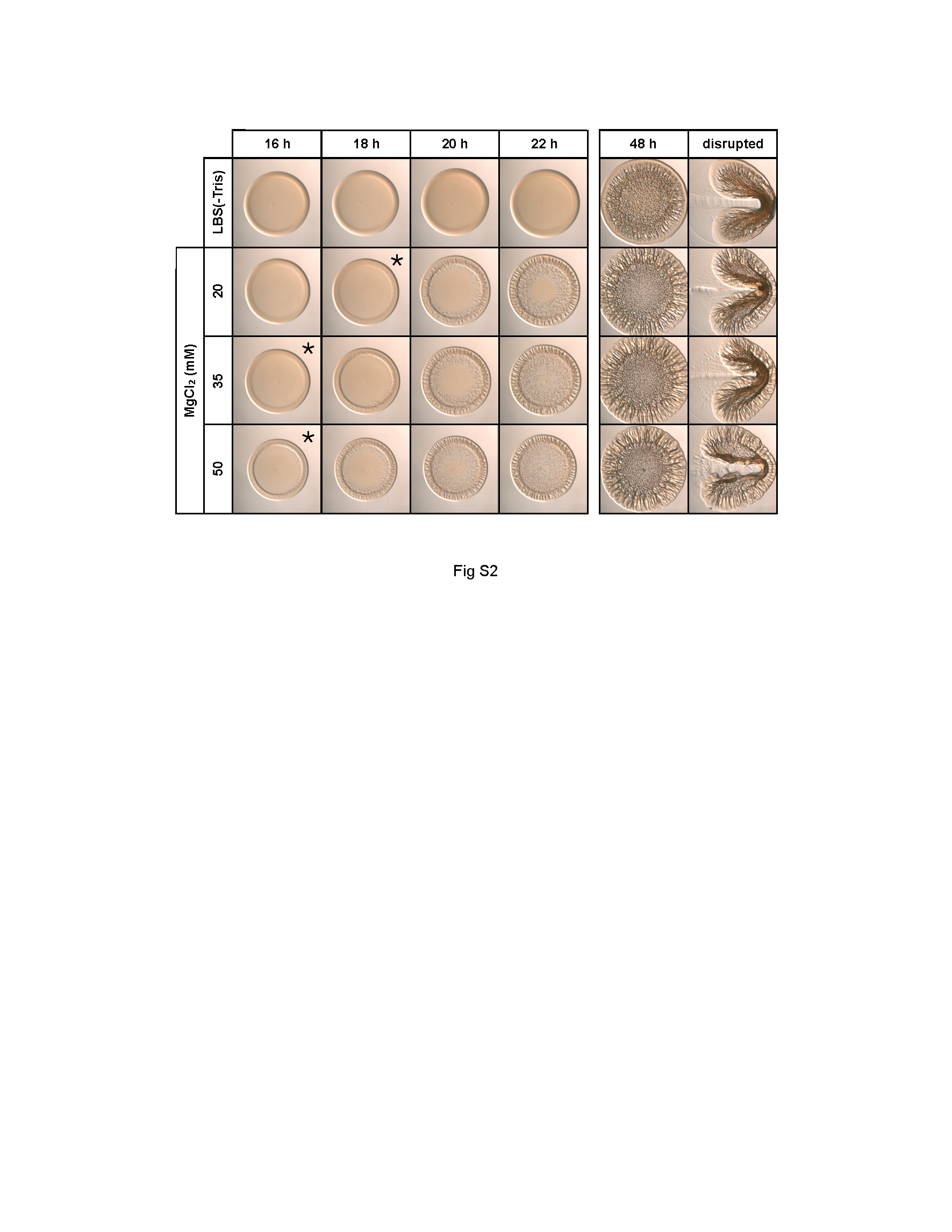

Supplement: S2 Fig — KV4366 was grown in LBS and spotted onto medium containing 1.0% tryptone, 0.5% yeast extract, 342 mM NaCl, and 0–50 mM KCl. Colonies were imaged at the same magnification at the indicated times and disturbed with a toothpick at the last time point. Asterisks indicate the first time point at which wrinkling became visible. (TIFF) [file pone.0169521.s002.tiff]

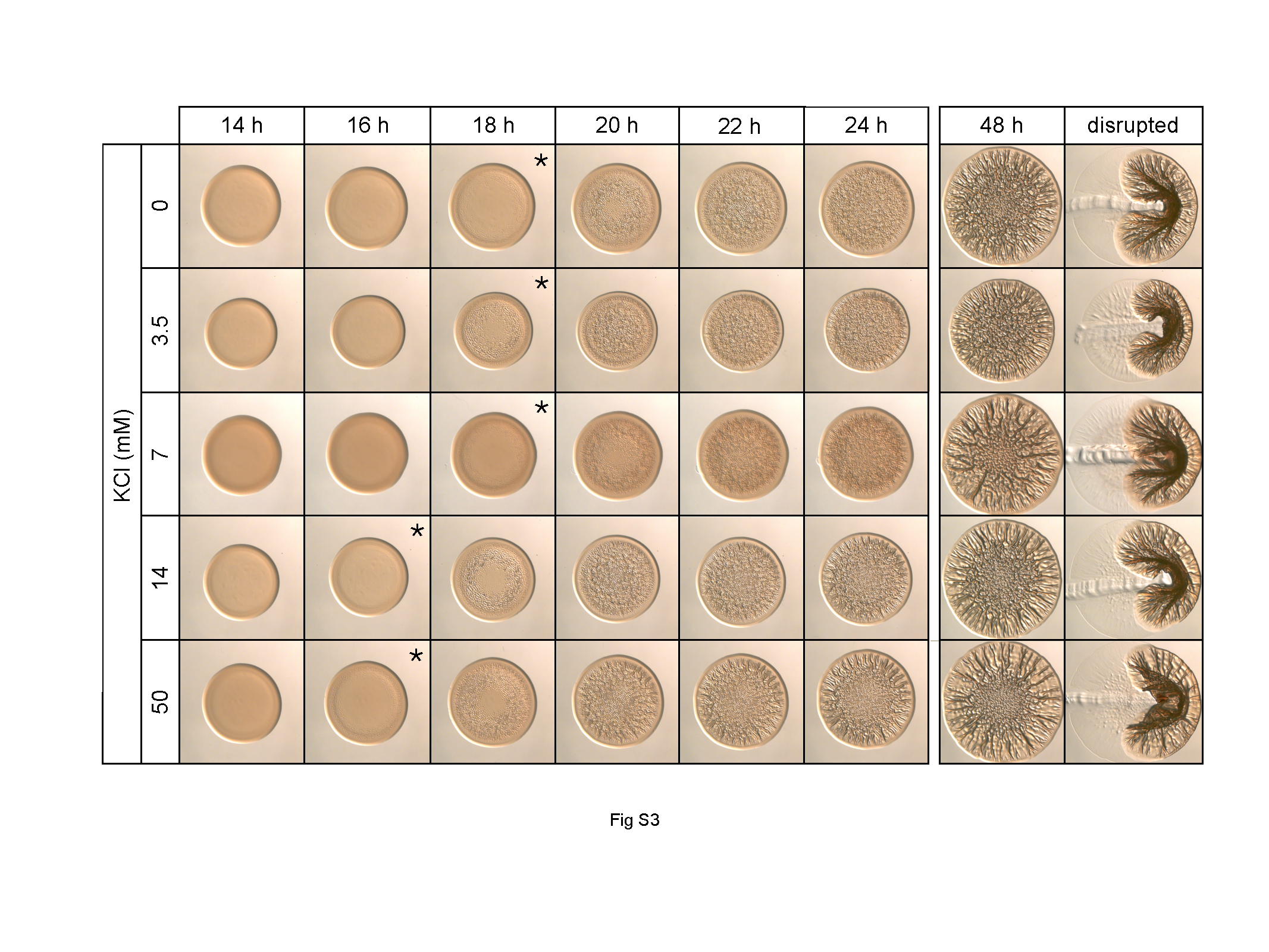

Supplement: S3 Fig — KV4366 was grown in LBS and spotted onto medium containing 1.0% tryptone, 0.5% yeast extract, 342 mM NaCl, and 0–50 mM KCl. Colonies were imaged at the same magnification at the indicated times and disturbed with a toothpick at the last time point. Asterisks indicate the first time point at which wrinkling became visible. (TIFF) [file pone.0169521.s003.tiff]

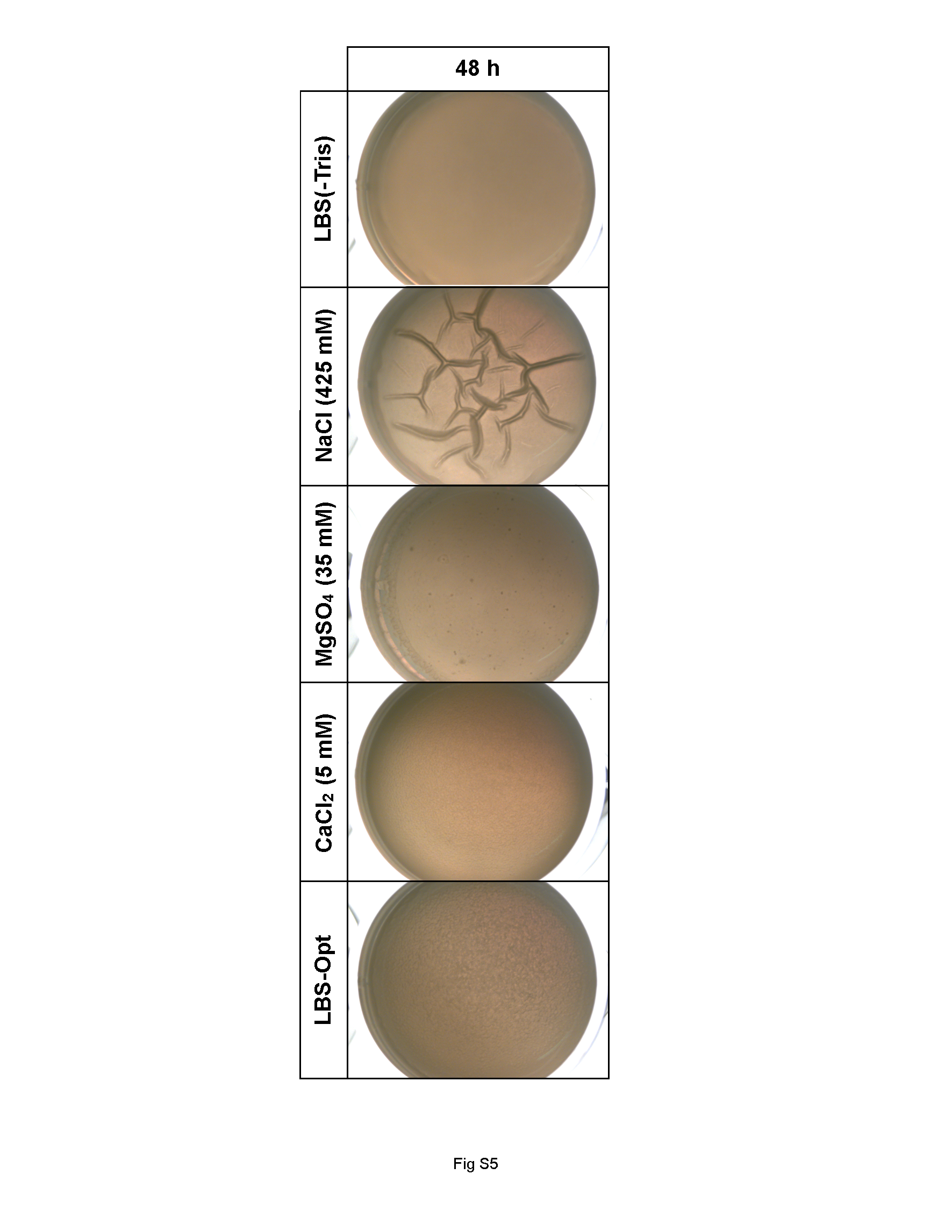

Supplement: S4 Fig — Plasmid-containing strains were grown in LBS and spotted onto LBS(-Tris), LBS-Opt, or LBS(-Tris) with 425 mM NaCl, 35 mM MgSO4, or 5 mM CaCl2. Strains carried either biofilm-inducing plasmids overproducing RscS (pKG11/ES114) (A) or SypG (pEAH73/ΔsypE) (B) or contained vector control plasmid pKV69 (pKV69/ES114 and pKV69/ΔsypE) (C and D, respectively). Images were captured at the same magnification after 24 or 48 h of growth at 24°C. Colonies were disturbed with a toothpick at the 48 hour time point. (TIFF) [file pone.0169521.s004.tiff]

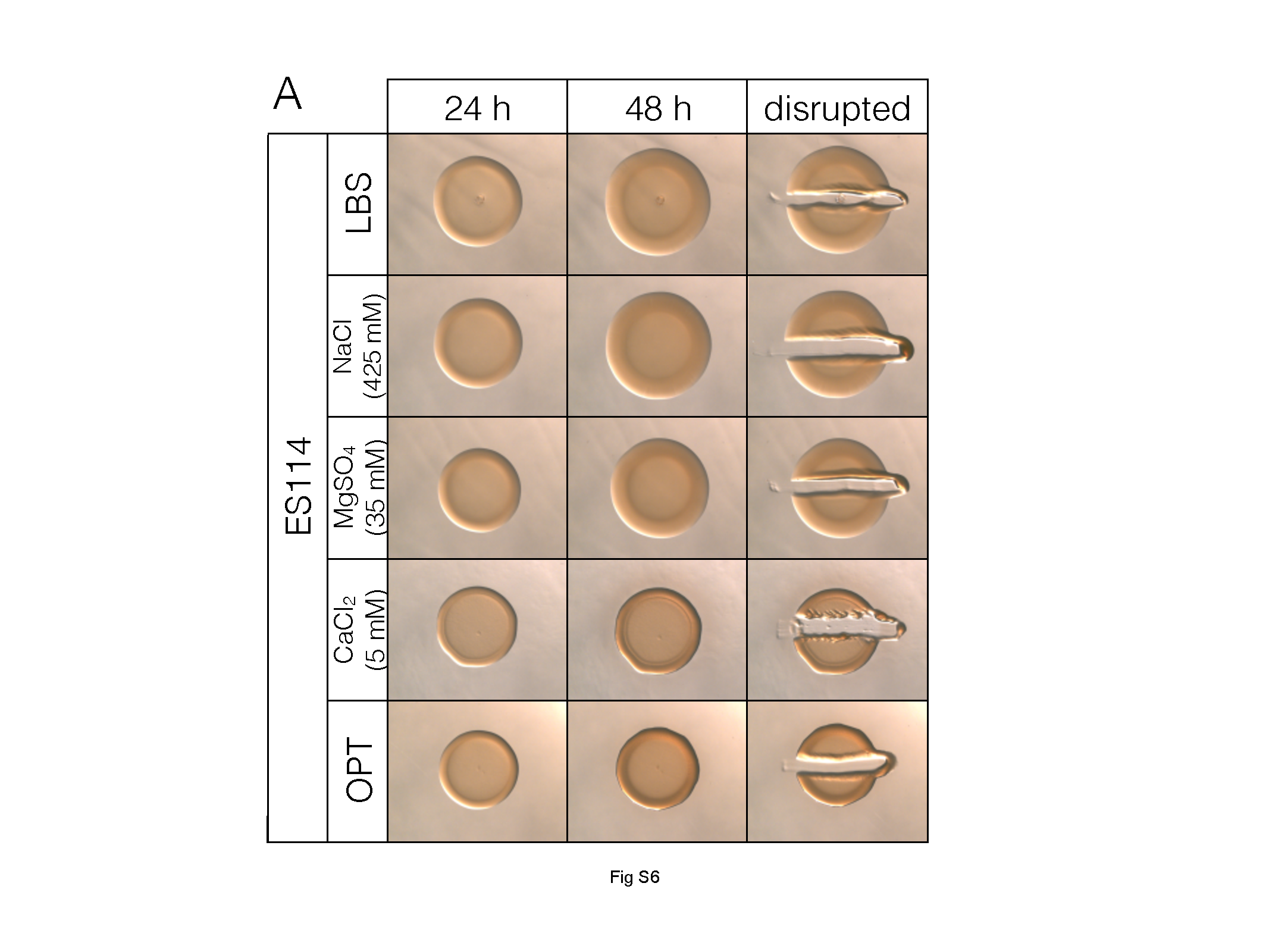

Supplement: S5 Fig — KV4366 was grown in LBS and diluted to an OD600 of 0.3 in LBS(-Tris) and LBS(-Tris) with 425 mM NaCl, 35 mM MgSO4, or 5 mM CaCl2, and LBS-Opt. Cultures were grown statically in the center wells of a 24-well plate at 24°C and imaged after 48 h. These images are the same as those in Fig 8 but are enlarged here to permit better visualization of the pellicle morphology. (TIFF) [file pone.0169521.s005.tiff]

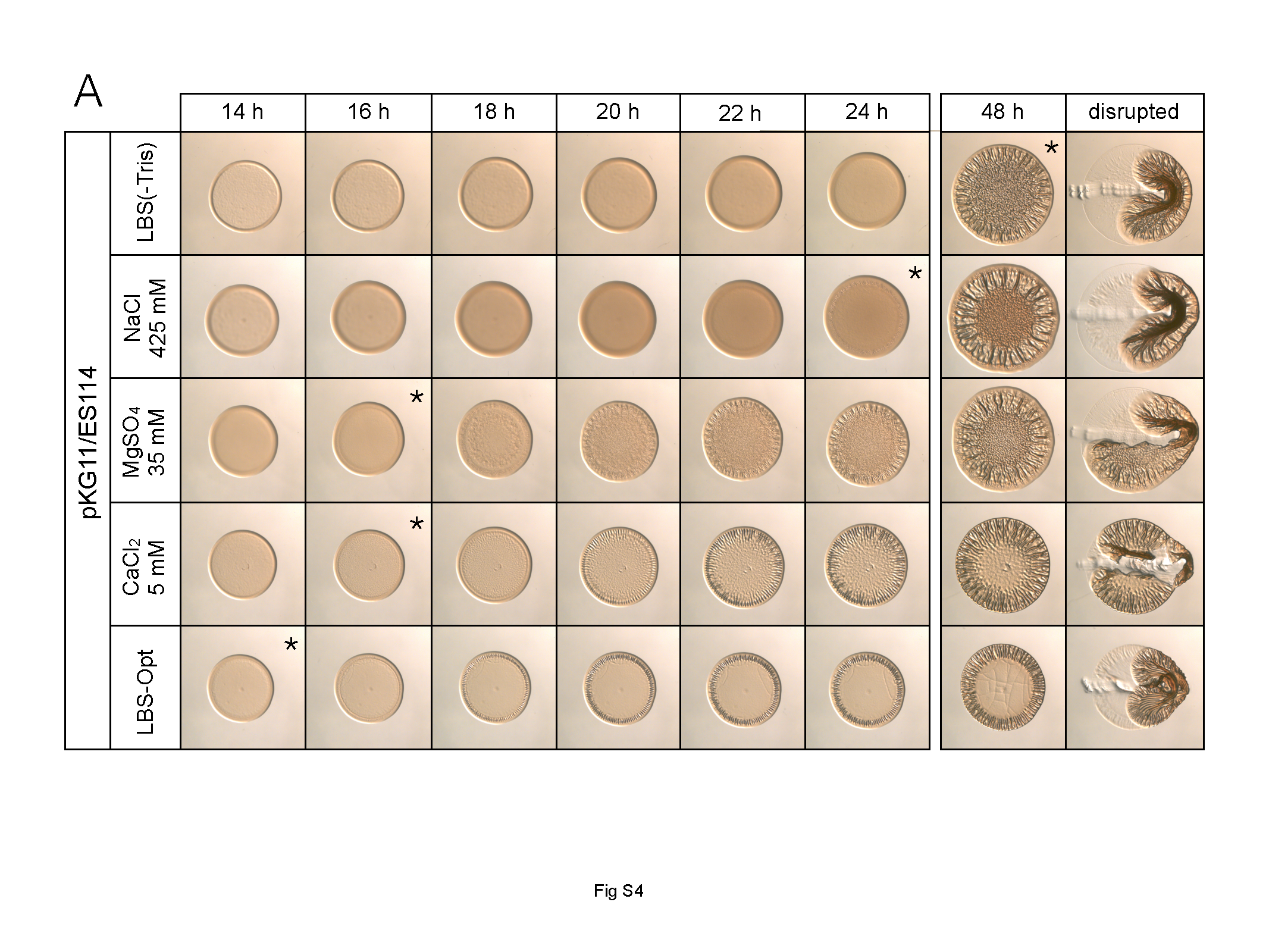

Supplement: S6 Fig — Wild-type V. fischeri strain ES114 was (A) grown in LBS and spotted onto LBS(-Tris) or LBS(-Tris) with 425 mM NaCl, 35 mM MgSO4, or 5 mM CaCl2, and LBS-Opt. Colonies were imaged at the same magnification after 24 or 48 h of growth at 24°C, and at the 48 hour time point colonies were disturbed with a toothpick. (B) ES114 was grown in LBS and diluted to an OD600 of 0.3 in LBS(-Tris) and LBS(-Tris) with 425 mM NaCl, 35 mM MgSO4, or 5 mM CaCl2, and LBS-Opt. Triplicate cultures were grown statically in the center wells of a 24-well plate at 24°C. After 20, 24, and 48 h, the surface of the liquid of each culture was disrupted with a toothpick and imaged; for each time point, images of the undisrupted and disrupted pellicles are presented from left to right. No pellicles were visualized. (TIFF) [file pone.0169521.s006.tiff]
